# Drug Resistance in Non-Hodgkin Lymphomas

**DOI:** 10.3390/ijms21062081

**Published:** 2020-03-18

**Authors:** Pavel Klener, Magdalena Klanova

**Affiliations:** 1 First Department of Internale Medicine-Hematology, University General Hospital in Prague, 128 08 Prague, Czech Republic; magdalena.klanova@vfn.cz; 2 Institute of Pathological Physiology, First Faculty of Medicine, Charles University, Prague, 128 53 Prague, Czech Republic

**Keywords:** non-Hodgkin lymphomas, drug resistance, chemotherapy, targeted agents

## Abstract

Non-Hodgkin lymphomas (NHL) are lymphoid tumors that arise by a complex process of malignant transformation of mature lymphocytes during various stages of differentiation. The WHO classification of NHL recognizes more than 90 nosological units with peculiar pathophysiology and prognosis. Since the end of the 20^th^ century, our increasing knowledge of the molecular biology of lymphoma subtypes led to the identification of novel druggable targets and subsequent testing and clinical approval of novel anti-lymphoma agents, which translated into significant improvement of patients’ outcome. Despite immense progress, our effort to control or even eradicate malignant lymphoma clones has been frequently hampered by the development of drug resistance with ensuing unmet medical need to cope with relapsed or treatment-refractory disease. A better understanding of the molecular mechanisms that underlie inherent or acquired drug resistance might lead to the design of more effective front-line treatment algorithms based on reliable predictive markers or personalized salvage therapy, tailored to overcome resistant clones, by targeting weak spots of lymphoma cells resistant to previous line(s) of therapy. This review focuses on the history and recent advances in our understanding of molecular mechanisms of resistance to genotoxic and targeted agents used in clinical practice for the therapy of NHL.

## 1. Introduction

Non-Hodgkin lymphomas comprise a heterogenous group of mature lymphoproliferative malignancies that arise as a result of malignant transformation during the process of lymphocyte differentiation and clonal expansion in secondary lymphoid organs [1,2]. Clinically, lymphomas comprise indolent, aggressive, and highly-aggressive malignancies with different treatment approaches and prognosis.

### 1.1. Genotoxic Agents and Their Combinations

From the dawn of anti-lymphoma therapy in the second half of the 20^th^ century, the prognosis of patients depended on our capability to classify the particular lymphoma subtype and deliver the most effective currently available treatment. The pioneer attempts to treat non-Hodgkin lymphomas (then lymphosarcomas) with single-agent cytostatics (e.g., alkylating agents—nitrogen mustard, cyclophosphamide or vinca alkaloids—vinblastine, vincristine) were associated with low response rates (predominantly partial remissions) and early relapses (usually within a few weeks) [3]. It soon became evident that combinations of cytostatic agents, namely cyclophosphamide and vincristine (Oncovin) (which together with prednisone formed a chemotherapy regimen called COP), were more effective compared to the respective monotherapies, not only because of additive anti-tumor activity of drugs A and B, but in the first place, because A plus B led to synthetic lethality with anti-lymphoma synergy capable of inducing first durable remissions in at least some lymphoma patients [4]. The discovery of anthracycline antibiotics in the early eighties and subsequent addition of hydroxydaunorubicin (doxorubicin) to COP resulted in the generation of the golden combination of genotoxic agents called CHOP, which have been used for the therapy of non-Hodgkin lymphomas up to the present [5]. Despite markedly improved outcomes achieved with CHOP, relapses were still frequent and the prognosis of relapsed patients dismal. Salvage regimens were usually based on diverse combinations of various genotoxic cytostatics, including platinum derivatives (cisplatin, carboplatin), alkylating agents (ifosfamide), epipodophyllotoxins (etoposide), and nucleoside analogues (cytarabine, gemcitabine, cladribine, fludarabine) [6,7,8].

### 1.2. Monoclonal Antibodies and Biological Therapy

In 1997, the first therapeutic monoclonal antibody (mAb) anti-CD20 rituximab was approved for the therapy of certain types of B-cell lymphomas [9,10,11,12]. Implementation of rituximab into CHOP (hence, R-CHOP) revolutionized the therapy of CD20+ B-NHL and soon, it was used not only as part of a front-line immunochemotherapy regimen, but also as a maintenance monotherapy in particular lymphoma subtypes [13,14,15,16]. Up to the present, R-CHOP remains the first-line standard of care for newly diagnosed patients with three most common types of B-NHL, diffuse large B-cell lymphoma (DLBCL), follicular lymphoma (FL), and marginal zone lymphoma (MZL) [17]. Despite great expectations and many attempts to introduce other mAbs targeting other antigens than CD20, rituximab and the next-generation anti-CD20 mAbs ofatumumab and obinutuzumab have remained, so far, the only naked therapeutic monoclonal antibodies approved for the therapy of B-NHL [9]. The mode of action of anti-CD20 mAbs is based in large part on the activation of complement (complement-dependent cytotoxicity) and host immune cells—natural killer cells or macrophages (antibody-dependent cell-mediated cytotoxicity), with minimal direct anti-lymphoma activity. Despite the fact that the natural anti-tumor immunity is mediated predominantly by T-lymphocytes, the first large-scale immunotherapy introduced in the clinical practice was based on monoclonal antibody-activated natural killer (NK) cells and macrophages. Consequently, the anti-lymphoma efficacy of mAbs can be impacted by compartment-specific differences caused not only by differences in the biodistribution of the antibody, but also by differences in subpopulations of macrophages in various compartments (e.g., in the bone marrow and lymph node masses), as well as by their functional status [18,19]. Recently, it was confirmed that chemotherapy-primed macrophages are significantly more effective mediators of rituximab-induced lymphoma cell elimination compared to chemotherapy naïve macrophages, which retrospectively provided rationale for the observed synthetic lethality between CHOP and rituximab [19].

### 1.3. Antibody-Drug Conjugates and Targeted-Drug Delivery

Antibody-drug conjugates (ADC) are mAbs with covalently-bound toxic payloads, usually anti-mitotic agents (e.g., monomethyl auristatin E (MMAE)) [20]. In contrast to therapeutic mAbs, the monoclonal antibody moiety in ADCs serves primarily for the targeted delivery of anti-tumor molecules to the tumor cells, while the immunologic functions of mAbs (complement-dependent cell-mediated cytotoxicity [CDC] and ADCC) are weakened. Up to the present, two ADCs have been approved for the therapy of NHL. Brentuximab-vedotin, anti-CD30 antibody with MMAE, was approved in 2011 for the therapy of relapsed or chemotherapy refractory (R/R) Hodgkin and anaplastic large cell lymphoma (ALCL), the two lymphoma subtypes characterized by aberrant expression of CD30 antigen [21,22]. Brentuximab-vedotin (alias SGN-35) was successfully developed, although the naked (therapeutic) anti-CD30 mAb (SGN-30) proved virtually ineffective in the same indication [23]. In 2017, brentuximab-vedotin was approved also for patients with cutaneous T-cell lymphomas [24]. Polatuzumab-vedotin, anti-CD79B antibody with MMAE, was approved in 2019 for the therapy of R/R DLBCL [25]. In addition, anti-CD22 ADCs inotuzumab-ozogamicin and moxetumomab-pasudotox were granted approval for patients with R/R B-cell acute lymphoblastic leukemia (2017), and R/R hairy cell leukemia (2018), respectively [26,27]. Several ADCs, e.g., anti-CD19 and anti-CD37, are currently in various stages of clinical development [28].

### 1.4. Signal Transduction Inhibitors and the Era of Targeted Therapy

Approval of the tyrosine-kinase inhibitor imatinib, the first signal transduction inhibitor approved in May 2001 for the treatment of chronic myelogenous leukemia, marked the beginning of the era of targeted therapy [29,30]. In 2013, the United States Federal Drug Administration (FDA) granted accelerated approval of ibrutinib, an inhibitor of Bruton tyrosine-kinase (BTK) and the first-in-class B-cell receptor (BCR) signaling inhibitor for the therapy of relapsed mantle cell lymphoma (MCL), chronic lymphocytic leukemia (CLL), and Waldenström macroglobulinemia (WM) [31,32]. In 2017, the second-generation BTK inhibitor acalabrutinib with limited off-target effects was approved for R/R MCL. Other next-generation BTK inhibitors (zanubrutinib, tirabrutinib) are in advanced clinical development [33]. Idelalisib was the first-in-class oral phosphatidylinositol 3-kinase (PI3K) inhibitor approved for the treatment of cancer, specifically for the therapy of relapsed CLL and FL [34]. In 2017, the FDA granted accelerated approval to copanlisib, an intravenous pan-class I PI3K inhibitor with predominant activity against the PI3K-α and -δ isoforms, in the therapy of R/R FL [35,36,37]. In 2018, the European Medicines Agency (EMEA) granted orphan designation to copanlisib for the treatment of R/R MZL. A second-generation PI3K-delta/gamma inhibitor, duvelisib, was approved for the therapy of CLL/SLL and FL after two or more systemic therapies [38,39]. Several other PI3K inhibitors (umbralisib, parsaclisib, buparlisib) are under various stages of preclinical and clinical development [40]. In addition to direct pharmacological inhibition with PI3K inhibitors and Src homology 2 containing inositol 5′ polyphosphatase 1 (SHIP1) activators, recently emerged as an effective novel therapeutic strategy to shut down aberrant PI3K–AKT signaling [41]. Temsirolimus, an inhibitor of the mammalian target of rapamycine (mTOR), a direct downstream client of PI3K–AKT signaling, was approved by the EMEA for the therapy of R/R MCL [42].

## 2. General Mechanisms of Development of Drug-Resistant Phenotype

Drug resistance has been associated with all types of anti-lymphoma therapy including genotoxic agents, mAbs, ADCs, targeted agents, or diverse drug combinations. The molecular mechanisms of drug resistance can be divided into cancer cell-intrinsic and cancer cell-extrinsic mechanisms. Inherent drug resistance is usually associated with preexisting factors that interfere with the mode of action of the anti-tumor agent (e.g., CD20-negative lymphomas will not respond to anti-CD20 rituximab). Acquired resistance is usually associated with measurable anti-tumor efficacy as a result of elimination of drug-sensitive lymphoma cells with ensuing temporary clinical remission of the disease. Subsequent relapse or progression of the lymphoma is therefore generally considered resistant to the drug(s) that induced the remission. 

### 2.1. Clonal Evolution Theory

**Figure 1:**
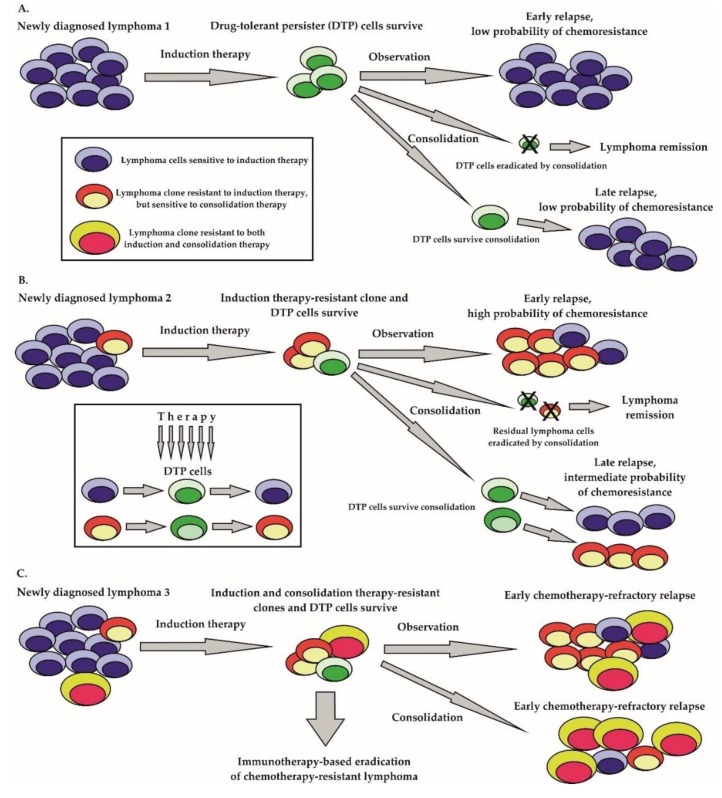
Resistance to therapy. **A**. Low level of genomic heterogeneity—lymphoma population without preexisting drug-resistant cells. **B**. Intermediate level of genomic heterogeneity—lymphoma population with the preexisting drug-resistant clone resistant to induction therapy; **C**. High-level of genomic heterogeneity—lymphoma population with several clones resistant to diverse immunochemotherapy regimens; DTP—drug-tolerant persister cells.

There are two major hypotheses by which cancer can recur after a previous (temporarily) effective therapy. According to the widely accepted clonal evolution concept, preexisting drug-resistant clones are selected by therapy, and sooner or later overgrow and replace the drug sensitive lymphoma population eliminated by the therapy (Figure 1B,C) [43,44,45]. The emergence of heterogeneous subclones within the originally clonal lymphoma population is believed to be a consequence of several key biological processes. Importantly, many lymphoma cells have an ongoing somatic hypermutation process, which facilitates the acquisition of additional genetic lesions [46]. Somatically mutated oncogenes, including enhancer of zeste 2 polycomb repressive complex 2 subunit (*EZH2*)*,* cAMP responsive element binding protein 1 (*CREBBP*)*,* innate immune signal transduction adaptor *MYD88*, immunoglobulin-associated gene *CD79B*, Notch receptor 1 (*NOTCH1*), transcription factor B-cell lymphoma 6 (*BCL6*) proto-oncogene serine-threonine kinase *PIM1*, or B-cell lymphoma 2 (*BCL2*) play important roles in the pathogenesis of aggressive lymphomas [47,48]. In addition, the class–switch recombination process required for the secretion of IgG molecules can lead to chromosomal translocations or gene deletions. The prototypical example is the translocation t(14;18), which results in the juxtaposition of the anti-apoptotic *BCL2* gene to the enhancer of transcription of heavy chains of immunoglobulin *IGHV* gene. The inactivation of key tumor suppressor genes by mutation or deletion (e.g., *TP53*, *ATM*, *CDKN2A, KMT2D*) contributes to a genomic instability that facilitates the acquisition of secondary mutations that can drive lymphoma evolution in a Darwinian fashion [49,50]. Lymphoma clones are selected by diverse pressures from within and from outside the tumor cells even before the initiation of anti-lymphoma therapy. Anti-tumor treatment that does not lead to lymphoma eradication in most instances results in the selection of drug-resistant disease. Complex comparative profiling of paired biopsies obtained from patients at diagnosis and at lymphoma relapse increased our knowledge of lymphoma evolution [51]. Apart from the clonal heterogeneity, the stochastic partitioning of cellular components can also contribute to the diversity of the lymphoma population [52]. 

### 2.2. Drug-Tolerant Persister Cells

Besides the generally accepted fact that tumor heterogeneity itself inevitably results in acquired drug-resistance, because it can be viewed as a reservoir of preexisting drug-resistant clones, single tumor cell plasticity can lead to adaptive transcriptional and post-transcriptional changes that can result in the derivation of the so-called “drug-tolerant persister” (DTP) cells (Figure 1A) [53]. It has been demonstrated that DTP cells exposed to anti-tumor drugs exploit adaptive mutability (a process similar to the increased mutation rate of bacteria exposed to antibiotics), which is achieved through the down-regulation of mismatch repair and homologous recombination genes with concomitant overexpression of error-prone polymerases [54]. In contrast to preexisting drug-resistant mutant clones, the drug-tolerant phenotype of DTP cells can be dynamically acquired and relinquished [55]. From the clinical point of view, both preexisting drug-resistant mutant clones and DTP cells correspond to minimal residual disease (MRD) in a patient on anti-cancer treatment. Both preexisting drug-resistant mutant clones and DTP cells can expand to establish a clinical relapse of the malignancy. Nevertheless, while preexisting mutant clones will outgrow to form a drug-resistant lymphoma, the DTP cells with transiently acquired drug-resistant phenotypes can establish a clinical relapse of predominantly drug-sensitive tumor cells (Figure 1).

### 2.3. Stem-Like Cells and Side-Populations Resistant to Therapy

Besides the clonal evolution, the stem-like cell theory proposes survival of lymphoma-initiating (stem-like) cells that possess inherent drug-resistant phenotypes or that can survive in specific niches in quiescent state [56]. Unlike solid tumors, our knowledge on potential lymphoma-initiating stem-like cells remains elusive [57]. Side population (SP) cells are defined by their ability to export Hoechst 33342 dye. SP cells express high levels of various members of the ATP-binding cassette **(**ABC) transporter family, including multi-drug resistance protein 1 (MDR1), which contribute to their drug resistant phenotype. SP populations are typically enriched in lymphoma-initiating cells characterized by increased self-renewal and clonogenicity in vivo. Candidate genes involved in lymphoma-initiating cell maintenance include for example B-cell specific Moloney murine leukemia virus integration site 1 (*BMI-1*) [58]. *BMI-1* was reported to be involved in self-renewal of cancer stem cells in various malignancies [58]. Genomic rearrangements of 10p12 leading to *BMI-1* gain were recurrently found in patients with the transformation of CLL into aggressive lymphomas (Richter transformation) and MCL [59,60]. MCL SP cells characterized by overexpression of BMI-1 possessed increased self-renewal capability and were highly tumorigenic in vivo [61]. Upregulation of BMI-1 in MCL cells leads to transcriptional repression of pro-apoptotic genes *BCL2L11/BIM* and *PMAIP/NOXA*, thereby fostering a drug-resistant phenotype of SP cells. Besides MCL, the upregulation of BMI-1 was associated with more adverse prognosis in FL [62]. Constitutive nuclear factor kappa B (NFκB) signaling resistant to bortezomib has been identified in a CD19-negative MCL-initiating stem-like cell population [63]. Oxidative stress, namely H_2_O_2_ has been demonstrated to promote lymphoma stemness phenotype, through the activation of the wingless (WNT)/β-catenin/MYC/sex determining region Y box 2 (SOX2) axis in the anaplastic lymphoma kinase-positive ALCL [64].

## 3. Cell-Intrinsic and Extrinsic Molecular Mechanisms of Drug Resistance

### 3.1. Cell-Intrinsic Mechanisms of Drug Resistance

**Figure 2:**
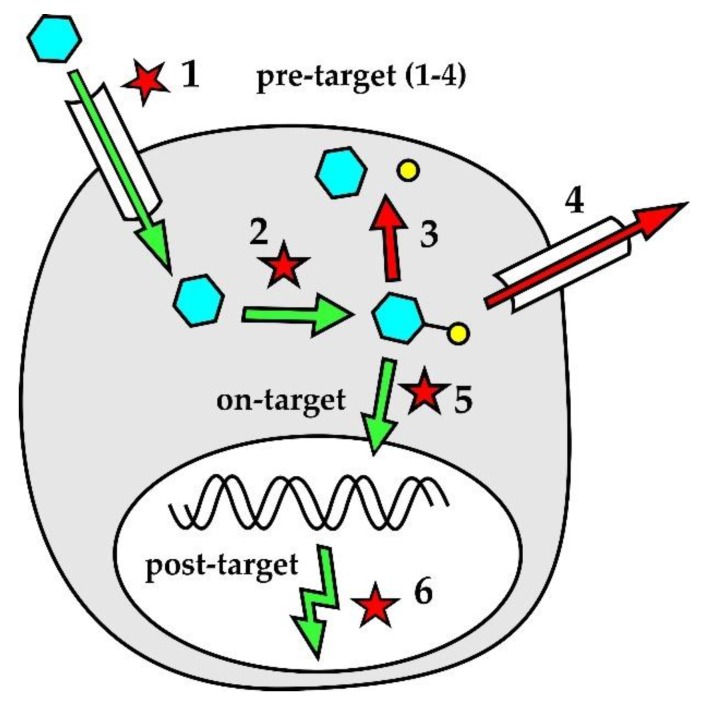
Cell-intrinsic mechanisms of drug resistance. 1. Inhibition of active drug transport within the lymphoma cell; 2. Inhibition of pro-drug activation into active metabolite(s); 3. Increased drug degradation; 4. Increased drug efflux; 5. Interference with drug mode of action, e.g., increased DNA repair; 6. Disruption of DNA damage response pathways.

Lymphoma cell-intrinsic molecular mechanisms of resistance to genotoxic agents are displayed in Figure 2. Pre-target mechanisms (Figure 2 [1,2,3,4]) include alterations of drug transport within the lymphoma cells and disruption of drug activation and/or inactivation pathways. Downregulation of human equilibrative nucleoside transporter-1 (hENT1), responsible for active transport of cytosine-arabinoside (ara-C) into leukemia and lymphoma cells, correlated with decreased sensitivity to ara-C. Both decreased expression of deoxycytidine-kinase (dCK) that phosphorylates ara-C prodrug into its active metabolite (ara-C-mono-phosphate, ara-CMP) and increased expression of cytidine-deaminase that dephosphorylates (deactivates) ara-CMP were repeatedly associated with resistance of leukemia or lymphoma cells to araC [65,66,67]. Increased expression of efflux pumps, namely multi-drug resistant protein 1 (MDRP-1) coding for a p-glycoprotein, was detected in patients with chemotherapy-resistant lymphomas [68]. On-target mechanisms of drug-resistance (Figure 2 [5]) interfere with the mode of action of genotoxic agents. Increased DNA repair machinery was associated with decreased drug efficacy. Post-target mechanisms of drug-resistance (Figure 2 [6]) comprise numerous defects of the DNA damage response pathway and apoptosis, including the inactivation of tumor suppressor *TP53* or aberrant overexpression of anti-apoptotic protein BCL2.

### 3.2. Cell-Extrinsic Mechanisms of Drug Resistance

**Figure 3:**
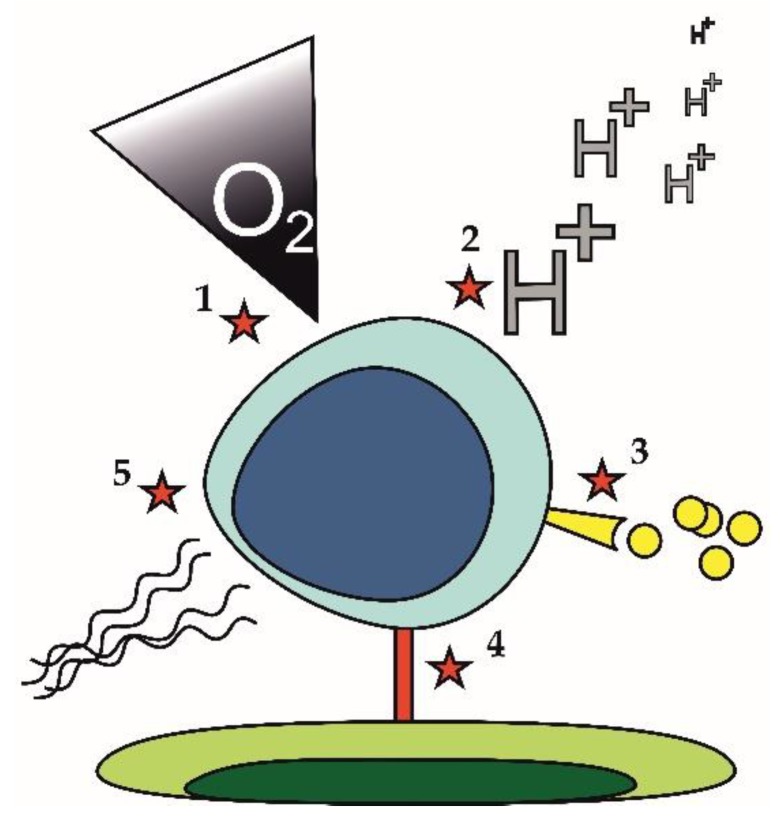
Cell-extrinsic mechanisms of drug resistance. 1. Hypoxia; 2. Acidosis; 3. Pro-survival growth factors and/or cytokines; 4. Cell-cell contact; 5. Alteration in the composition of extra-cellular matrix.

Lymphoma cell-extrinsic mechanisms that may contribute to the development of the drug-resistant phenotype are displayed in Figure 3. They comprise hypoxia and acidosis (Figure 3 [1,2]), which may trigger metabolic rewiring of lymphoma cells. Hypoxia and accompanying acidosis can cause resistance of lymphoma cells in a very complex way described in detail in a separate section of this review (Section 3.4). Increased secretion of pro-survival cytokines or growth factors (Figure 3 [3]) was also repeatedly associated with drug resistance. Increased secretion of interleukine 6 (IL6) was responsible for acquired resistance of lymphoma cells to PI3K inhibitors. Cell to cell contact (Figure 3 [4]) can induce drug resistance through increased expression of anti-apoptotic molecules. Upregulation of BCL-XL upon binding of leukemia or lymphoma cells to fibroblasts expressing CD40 ligand conferred resistance to venetoclax [69]. Changes in the composition of extracellular matrix (Figure 3 [5]), e.g., increased deposition of collagen fibers leading to fibrotization, can impact drug delivery to tumor tissue thereby fostering drug resistance [70].

### 3.3. Compartmentalization of Lymphoma Cells and Survival of Anti-Lymphoma Therapy

Lymphoma cells do not necessarily have to acquire specific drug-resistant phenotypes to be able to survive anti-lymphoma therapy. A typical example is the involvement of the central nervous system (CNS) or other immune-privileged sites [71]. Thanks to the hemato-encephalic barrier the CNS compartment is not exposed to effective plasma levels of standard front-line anti-lymphoma regimen (e.g., R-CHOP), thereby enabling lymphoma cell survival with no need for large-scale genomic, transcriptional, or post-translational changes. In analogy, bulky lymphoma masses with extensive areas of necrotic tissue may enable survival of lymphoma cells that are not exposed to effective levels of anti-lymphoma drugs because of defective drug delivery. This can be at least partially overcome by new formulations of old cytostatic agents, e.g., by encapsulation of small molecule chemotherapy agents in liposomes. The enhanced permeability and retention (EPR) effect results in passive trapping of large liposomes within the chaotic lymphoma vasculature with prolonged exposure of tumor cells to the toxic payloads, while (relatively) sparing healthy tissues [72]. However, in contrast to other hematologic malignancies including acute myelogeneous leukemia or multiple myeloma, liposomal formulations of anthracyclines or cytarabine have not yet been approved for the therapy of NHLs [73].

### 3.4. Hypoxia-Induced Changes and Metabolic Reprogramming 

**Figure 4:**
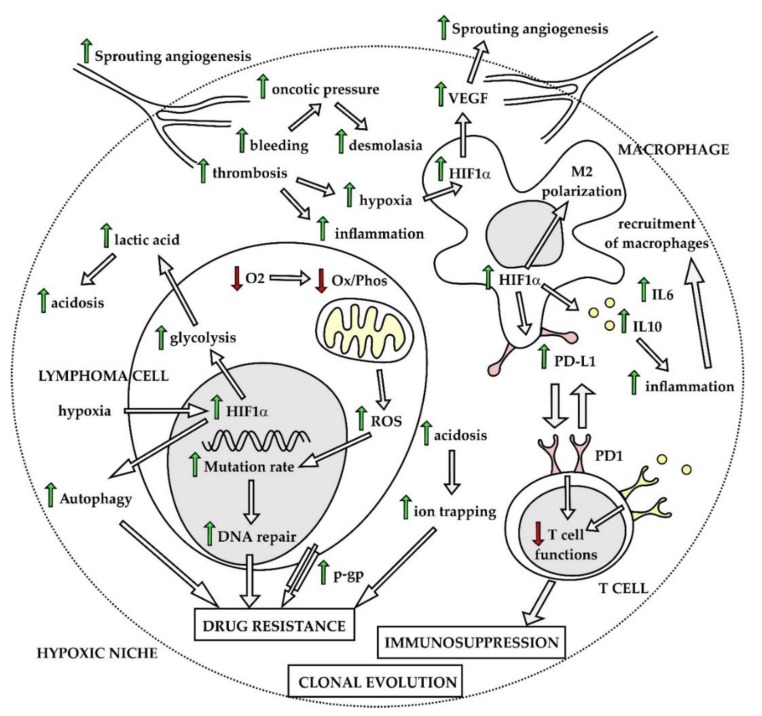
Hypoxic niche. Ox/Phos—oxidative phosphorylation, HIF1—hypoxia-inducible factor 1, PD1—programmed death 1, PD-L1—PD ligand 1, VEGF—vascular endothelial growth factor, O_2_—oxygen, ROS—reactive oxygen species.

Lymphoma cells often survive and proliferate in highly hypoxic microenvironments including bulky necrotic lymphoma masses, malignant effusions, but also bone marrow [74]. To be able to grow and spread, lymphoma cells must learn how to overcome stress-induced by hypoxia, acidosis, increased levels of reactive oxygen species (ROS), and lack of nutrients. Hypoxia belongs to widely recognized factors that in a very complex fashion facilitate tumor cell survival and chemoresistance in all types of malignant diseases [75]. Hypoxia-inducible factors (HIFs) play a central role in the string of adaptation phenotypic changes associated with hypoxia, both in lymphoma cells and non-malignant cells of the tumor microenvironment [76]. Vascular endothelial growth factor (VEGF) transactivated predominantly by HIF1α triggers sprouting angiogenesis, and functions as an autocrine and paracrine growth factor, for the lymphoma and non-malignant cells of the tumor microenvironment, respectively. Chaotic neovascularization of blood vessels leads to repeated thrombotic and hemorrhagic events, while lack of organized lymphatic vessels results in increased oncotic pressure of intercellular space, which facilitates deposition of collagen fibers. Hypoxia-induced polarization of tissue-residing macrophages triggers secretion of pro-inflammatory cytokines and recruitment of non-malignant cells of the immune system. Hypoxic, fibrotic, and pro-inflammatory niches next to areas of necrotic tissues together form permissive microenvironment for the lymphoma cell survival and development of drug-resistance (Figure 4). Hypoxia leads to decreased pH of the tumor microenvironment predominantly through increased lactic acid production as a result of enhanced glycolysis. Acidosis was shown to contribute to chemoresistance by inhibition of host immune functions and by decreasing activity of several cytostatic agents [77]. Non-malignant cells of the tumor ecosystem including tumor-associated macrophages (TAMs), myeloid-derived suppressor cells or subpopulations of T lymphocytes also undergo hypoxia-induced phenotypic changes that critically contribute to the survival of lymphoma cells by fostering immunosuppression, by aberrant expression of programmed death ligand 1 and by secreting immunosuppressive cytokines, e.g., IL6 or IL10 [78]. Cellular stress induced by hypoxia and lack of nutrients can activate autophagy, which was repeatedly associated with the drug-resistant phenotype [79]. 

Another important consequence of hypoxia is metabolic reprogramming of cancer cells [80]. Enhanced glycolysis, an observation first described by Otto Warburg in 1956, belongs to widely accepted hallmarks of cancer [81,82]. The reasons for the observed metabolic switch toward increased anaerobic and aerobic glycolysis remain to be fully elucidated. It is plausible that glycolysis allows for rapid production of ATP together with generation of intermediate metabolites for de novo synthesis of nucleotides, which is critical for lymphoma cells with high mitotic activity. Metabolic reprogramming is regulated by HIF-1alpha and fostered by diverse oncogenic signaling pathways, including aberrant activation of the PI3K–AKT–mTOR pathway, activation of oncogenes (e.g., *MYC*, *NFkappaB*), or loss of tumor suppressors (e.g., phosphatase and tensin homolog, *PTEN*, *TP53*, *CDKN2A*), recurrently observed in malignant lymphomas [83]. In lymphoma cells, the metabolic reprogramming is mainly mediated by mTOR complex 1, which conveys key signals from the microenvironment (glucose levels, growth factors, and tension of oxygen) to basal cell energy/metabolic pathways including oxidative phosphorylation, glutaminolysis, tricarboxylic acid (TCA) cycle, pentose phosphate pathway (PPP), lipid synthesis, and aerobic glycolysis. The MTORC1 complex also belongs to key regulators of autophagy. Besides mTORC1, AMP-activated protein kinase (AMPK) belongs to critical regulators of the metabolic adaptation process via redox regulation, specifically by maintaining sufficient NADPH levels under hypoxia and glucose limitations, which is critical for tumor cell survival under stress conditions [84]. In addition to cancer cells, non-malignant cells of the tumor ecosystem (macrophages, T-cells) have been shown to undergo similar metabolic reprogramming with so far unknown consequences on their crosstalk with the tumor cells. 

Metabolic reprogramming in different types of NHL is still a matter of investigation. Transcriptional profiling of DLBCL revealed three different categories: 1, “ox/phos” cluster enriched in genes involved in mitochondrial metabolism, 2, “BCR” cluster associated with glycolysis, and 3, “host-response (HR)” cluster enriched in transcription of genes regulating T-cell responses [85]. Recently, it was demonstrated that lymphomas with low expression of GAPDH use predominantly the ox/phos metabolism and rely on mTORC1 signaling and glutaminolysis for ATP generation [86]. Glutamine represents an important nutrient essential for proliferation of lymphoma cells under hypoxic condition and deprivation of glucose [87]. Gene set enrichment analysis of mantle cell lymphoma lines cultured in vitro and grown in vivo as lymphoma xenografts in immunodeficient mice revealed that genes involved in ox/phos represented the most significantly changed category, and that the changes were fully reversible by ex vivo culture of lymphoma xenografts [88]. The metabolic state of the studied lymphoma cells thus demonstrated large plasticity and reflected differences in in vitro and in vivo conditions, in which lymphoma cells proliferated. 

Taken together, changes in basal cell energy/metabolic pathways among diverse tissue compartments with various levels of hypoxia, as well as vast differences in metabolic demands between quiescent and proliferating lymphoma cells, have huge impacts on responses to various anti-cancer treatments and play important roles in survival, clonal evolution, and development of drug resistance.

## 4. General Approaches to Prevent Recurrence of Drug-Resistant Lymphomas

### 4.1. Drug Combinations and Sequential Therapy

There are two effective approaches that diminish or even prevent recurrence of drug-resistant lymphomas. The historically older approach was based on effective drug combinations (compared with monotherapies) that induced cytotoxic synergy and potentially targeted different lymphoma subclones or effectively prevented the survival of DTP cells. The recurrence of lymphomas even after a combined immunochemotherapy regimen led to the design and clinical testing of various sequential protocols that typically comprised an induction phase, a consolidation phase, and, in some lymphoma subtypes, also a maintenance phase. Drug combinations and sequential therapy with diverse drug combinations thus represent two empirical concepts to prevent the recurrence of drug-resistant lymphomas. 

### 4.2. “General” Drug Sensitizers

Several attempts have been undertaken to overcome the drug-resistant phenotype of lymphoma cells with currently approved agents. Verapamil functions as a p-glycoprotein inhibitor and it was tested in combination with chemotherapy in several trials in patients with aggressive lymphomas, but the results were rather disappointing [68,89,90]. Proton pump inhibitors modulate highly acidotic tumor microenvironments responsible for the inactivation (protonation) of the majority of conventional anti-tumor drugs (weak bases) by increasing the pH of the cancer ecosystem [91]. Chloroquine and hydroxychloroquine have been used in combination therapies in order to overcome stress-induced autophagy responsible for lymphoma cell survival [92]. A broad range of diverse anti-tumor activities were associated with an anti-diabetic biguanide drug, metformin. The anti-lymphoma efficacy of metformin, however, remains controversial. On one side, it was reported that treatment with metformin in patients with type 2 diabetes was associated both with a lower risk of lymphoma incidence and with a better outcome for patients with lymphoma [93,94]. In contrast, metformin use was not associated with improved outcome in newly diagnosed DLBCL and FL in a recently published large retrospective analysis [95].

### 4.3. Immunotherapy Approaches

Some types of relapsed lymphomas cannot be cured with any currently available immunochemotherapy or targeted salvage regimen. In these cases, the only potentially curative approach includes effective lymphoma debulking (by salvage therapy) followed by consolidation with allogeneic stem cell transplantation (alloSCT). AlloSCT, a form of adoptive T cell-based immunotherapy, can induce durable remissions in the patients who respond to salvage therapy, by graft-versus-lymphoma-mediated eradication of tissue-residing lymphoma-initiating quiescent cells, irrespective of which molecular/cytogenetic lesions they might harbor. AlloSCT will be, probably in the near future, replaced by ex vivo expansion and re-administration of genetically engineered autologous T cells expressing a so-called chimeric antigen receptor (CAR T-cells) capable of targeting residual lymphoma cells with no need for co-stimulation, a new generation bi-specific (or tri-specific) T-cell engaging monoclonal antibodies, or other forms of T cell-based immunotherapy, e.g., immune check-point inhibitors. These novel immunotherapy approaches based on genetically engineered cells and monoclonal antibody constructs have been coined synthetic immunity as opposed to both natural and adaptive immunity.

## 5. Genotoxic Agents: DNA Damage Response and Induction of Mitochondrial Apoptosis

### 5.1. Resistance to Alkylating Agents

Alkylating agents and other classical cytostatics, e.g., platinum derivatives or anthracyclines, exert their anti-tumor activity by disrupting DNA replication, which activates DNA damage response (DDR) pathways including triggering of mitochondrial apoptosis. Classical cytostatics are the prototypical anti-proliferative chemotherapy agents that target rapidly dividing lymphoma cells, which are associated with broad-range activity toward the tumor, but also with substantial toxicity toward healthy tissues. Molecular mechanisms associated with resistance to alkylating agents appear to be complex. They comprise both intrinsic and extrinsic mechanisms including various pre-target (e.g., overexpression of p-glycoprotein and other efflux pumps), on-target (e.g., defects or aberrant activation of DNA repair mechanisms), and post-target mechanisms (e.g., defects mitochondrial apoptosis or stress-induced autophagy), as well as diverse microenvironmental factors [68,91,96,97]. 

#### 5.1.1. DNA Damage Response and Disruption of Mitochondrial Apoptosis

**Figure 5:**
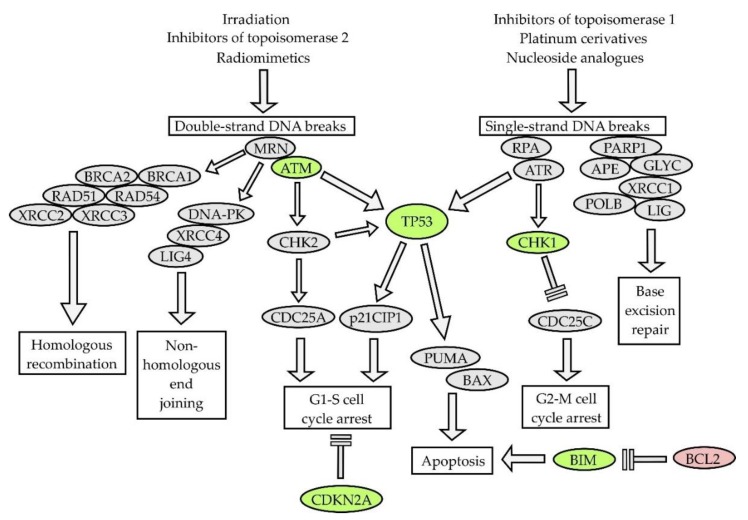
DNA damage response pathways. Double-strand DNA breaks: MRN—MRE11–RAD50–NBN (Nibrin) complex, ATM—ataxia-teleangiectasia mutated, BRCA1, BRCA2—breast-related cancer antigen 1, 2, XRCC2/3—X-ray repair cross complementing 2/3, DNA-PK—DNA protein-kinase, XRCC4—X-ray repair cross complementing 4, LIG4—DNA ligase 4, CHK2—check-point kinase 2, CDC25—cell division cycle 25; Single-strand DNA breaks: RPA—replication protein A, ATR—ataxia teleangiectasia and RAD3 related, CHK1—check-point kinase 1, CDC25C—cell division cycle 25C, PARP1—poly(ADP-ribose) polymerase 1, APE—exonuclease III APE, GLYC—DNA glycosylase, XRCC1—X-ray repair cross complementing 1, LIG—DNA ligase, POLB—DNA polymerase beta. Green—tumor suppressor genes recurrently mutated in lymphomas ATM, TP53, CHK1, CDKN2, red—oncogene BCL2 (B cell lymphoma 2) frequently overexpressed in lymphomas.

The mode of action of classical cytostatic agents is mediated mainly through DNA-damage response (DDR) pathways and affects predominantly rapidly dividing lymphoma cells (Figure 5). DDR pathways are triggered in response to various types of DNA damage including single- and double-strand DNA breaks, inter-strand cross-links, or stalking of the replication fork [98]. Germ line mutations of genes that regulate DDR pathways have been associated with diverse inherited cancer syndromes (e.g., ataxia teleangiectasia, Li–Fraumeni syndrome, etc.). It is not surprising that key tumor suppressor genes that control DDR are structurally or functionally deregulated in tumors, and that such aberrations are acquired or selected following chemotherapy. On the other hand, aberrant activation of DDR can decrease the efficacy of anti-cancer therapy by helping the cancer cells to survive the elevated replication stress. Targeted inhibition of selected DDR pathways in cancer cells, e.g., with the poly(ADP)ribose-polymerase 1 (PARP1) inhibitor olaparib or check-point kinase 1 (CHK1) inhibitors, may induce synthetic lethality with chemotherapy. Parvin et al. demonstrated that DLBCL cells expressing the LIM2 domain-only 2 (LMO2) protein are functionally deficient in homologous recombination-mediated DNA double-strand break repair and exhibit high sensitivity to PARP1 inhibitors [95]. Some of these approaches are currently tested in several clinical trials in patients with various types of NHL. 

Aberrations of *TP53* gene can be found in approximately 40% of T-NHL, 20% of DLBCL, Burkitt lymphoma, MCL, and MZL, but only in 5%–10% of FL [99]. Aberrations of *TP53* predicted poor outcome across all lymphoma subtypes. Transcription factor p53 directly transactivates pro-apoptotic genes *PUMA* and *NOXA* and indirectly induces the stabilization of anti-apoptotic BCL2 (through the downregulation of micro RNAs miR15a and miR16-1, both of which are also transactivated by p53). Inactivation of *TP53* is therefore associated with complex defects in mitochondrial apoptosis. Aberrations of ataxia-teleangiectasia mutated (*ATM,* deletions or mutations) can be found in approximately 40% patients with MCL, but they have never been associated with clinical outcome [100]. Cyclin-dependent kinase inhibitor *CDKN2A* codes for two tumor suppressor proteins, a cyclin-dependent kinase 4/6 inhibitor p16INK4A, and p14ARF, which is responsible for the induction of apoptosis in response to the overexpression of oncogenes (e.g., *MYC*). Inactivation of *CDKN2A* as a result of chromosome 9p deletion has been found in approximately 20% of MCL, and DLBCL, and is associated with adverse outcome, chemoresistance, and CNS involvement or relapse [101,102,103].

Deregulation of programmed cell death is another hallmark of lymphoma. Both variable/(diversity)/joining (V(D)J) immunoglobulin gene recombination implemented by recombination activating genes 1 and 2, and somatic hypermutation and class–switch recombination, both implemented by activation-induced deaminase, are events that specifically occur in lymphocytes during various stages of maturation and that, in a targeted manner, disrupt the integrity of DNA, which can result in (erroneous) mutation of non-Ig genes including chromosomal translocations and somatic mutations. A translocation t(14;18), which results in juxtaposition of the (entire) anti-apoptotic *BCL2* gene to the transcriptional enhancers of heavy chain immunoglobulin loci, can be found in >90% of FL and in approximately 20% of DLBCL [104]. Besides the canonical translocation t(14;18), BCL2 protein overexpression can be caused by *BCL2* gene gain/amplification. In addition, aberrant BCL2 expression can be at least partially fostered by deletion of chromosome 13q14.2 region coding for micro RNAs miR15a and miR16-1 that bind to and degrade BCL2 mRNA. The deletion of the *TP53* gene, which directly transactivates these micro RNAs, also leads to the stabilization of BCL2. These mechanisms (i.e., *BCL2* gains, *TP53* aberrations, and 13q14.2 deletions) contribute to BCL2 protein overexpression in virtually all MCL and lymphoplasmacytic lymphomas. Importantly, BCL2 is not only directly involved in the pathogenesis of NHL, but also in inherent or acquired drug resistance. Venetoclax (ABT-199/GDC0199) was the first-in-class BCL2 inhibitor approved for the therapy of chronic lymphocytic leukemia, and, recently, of acute myeloid leukemia (see Section 9). Not all lymphoma subtypes, however, rely on BCL2 antiapoptotic signaling for survival. We have demonstrated that BCL2-negative DLBCL that do not express the BCL2 protein by standard immunohistochemistry rely on MCL1 expression for survival [105]. Moreover, the combined inhibition of BCL2 and MCL1 led to synthetic lethality both in DLBCL and MCL [105,106]. In addition to BCL2 and MCL1, disruption of other key players or regulators of apoptotic pathways have been associated with poor clinical outcome (e.g., BCL-XL, BIM, and BIRC3) [69]. Low protein expression of BIM correlated with adverse outcome in MCL [107]. Loss-of-function mutations or gene deletions of *BIRC3* correlated with resistance to fludarabine [108].

#### 5.1.2. Stress-Induced Autophagy and Drug-Resistant Phenotype

Autophagy is a catabolic process of recycling of damaged organelles and other cellular components through lysosomal degradation [92]. Under normal conditions, the level of autophagy is generally low. Under various stress conditions (lack of nutrients, hypoxia, activation of oncogenes, and inactivation of tumor suppressors), autophagy is activated to facilitate survival. Stress-induced activation of autophagy under hypoxia, lack of nutrients, or upon chemotherapy administration was repeatedly associated with chemoresistance [109]. As a consequence, inhibitors of autophagy have been tested to overcome the drug-resistant phenotype of cancer cells, including lymphomas [110]. Hypoxia-induced autophagy was reported to contribute to decreased chemosensitivity in T-cell lymphomas [111]. It was demonstrated that the hypoxia- and IL6-mediated activation of autophagy promotes MCL cell survival and chemoresistance, and that the inhibition of autophagy overcomes the resistance to mTOR inhibitors [112,113,114]. 

### 5.2. Resistance to Nucleoside Analogues

**Figure 6:**
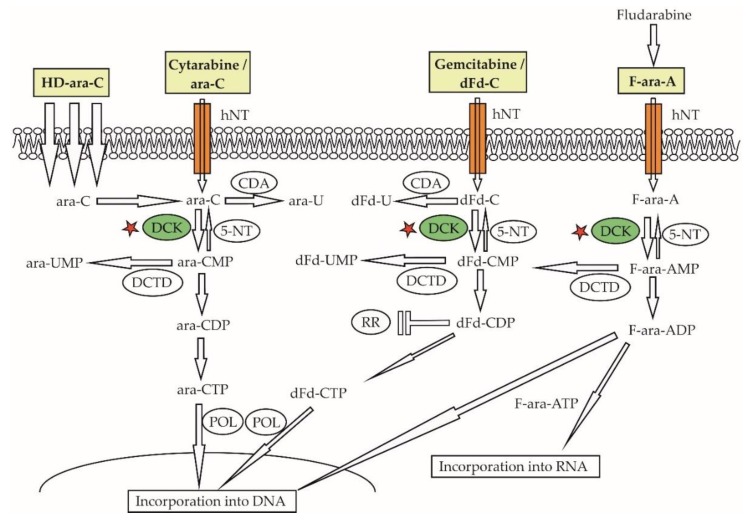
Downregulation of deoxycytidine-kinase confers resistance to a broad range of antinucleosides. HD-ara-C—high-dose cytarabine, ara-C—cytarabine, ara-CDP—Arabinofuranosylcytosine-diphosphate, ara-CMP—Arabinofuranosylcytosine-monophosphate, ara-CTP—Arabinofuranosylcytosine-trisphosphate, CDA—cytidine-deaminase, DCK—deoxycytidine-kinase, DCTD—deoxycytidine-monophosphate deaminase, dFd-C—gemcitabine, dFd-CDP—Difluorodeoxycytidine-diphosphate, dFd-CMP—Difluorodeoxycytidine-monophosphate, dFd-CTP—Difluorodeoxycytidine-triphosphate, F-ara-A—fludarabine, F-ara-AMP—9-β-D-arabinofuranosyl-2-fluoroadenine-monophosphate, F-ara-ADP—9-β-D-arabinofuranosyl-2-fluoroadenine-diphosphate, F-ara-ATP—9-β-D-arabinofuranosyl-2-fluoroadenine-triphophate, hNT—human nucleotide transporters, POL—DNA polymerase, 5-NT—5-nucleotidase.

Nucleoside analogues belong to the oldest genotoxic agents used in the therapy of malignant lymphomas. Ara-C, a backbone anti-leukemic drug, has been used for decades for salvage therapy of malignant lymphomas, as part of diverse salvage chemotherapy regimen. Ara-C is a prodrug that must be transported within the cancer cells, phosphorylated by deoxycytidine-kinase (DCK) and other kinases to cytarabine-tris-phosphate (ara-CTP), and incorporated into DNA, to be able to exert its anti-proliferative activity (Figure 6) [115]. Relatively higher levels of the cytarabine transporter SLC29A1 mRNA and its protein human equilibrative nucleoside transporter-1 (hENT1) observed in MCL compared with CLL cells might be at least partially responsible for the overall good sensitivity of MCL cells to araC [116]. Indeed, the addition of high-dose ara-C to chemotherapy has become a standard of care for upfront therapy of transplant-eligible patients with MCL [117]. We have demonstrated that acquired resistance of MCL cells to ara-C was caused by downregulation of the DCK protein [67]. As a consequence, acquired araC resistance was associated with “cross”-resistance to all nucleoside analogues that require activation via DCK phosphorylation including fludarabine, gemcitabine, and cladribine. In analogy, the downregulation of DCK observed in MCL cells with acquired resistance to fludarabine was associated with “cross”-resistance to ara-C, gemcitabine, and cladribine [118]. Freiburghaus et al. recently confirmed that the downregulation of DCK belongs to key molecular events responsible for ara-C resistance and reported that it can be prevented by treatment with the proteasome inhibitor bortezomib [119]. The downregulation of SLC29A1/hENT1 ara-C transporter and upregulation of cytidine-deaminase (that inactivates ara-C to an inactive metabolite) were detected in ara-C-resistant AML cells [65,66]. Mutations of the anti-apoptotic gene *BIRC3* and splicing factor *SF3B1* have correlated with fludarabine refractoriness in chronic lymphocytic leukemia/small lymphocytic lymphoma [108,120].

## 6. Resistance to Ibrutinib and other Bruton Tyrosine-Kinase Inhibitors

**Figure 7:**
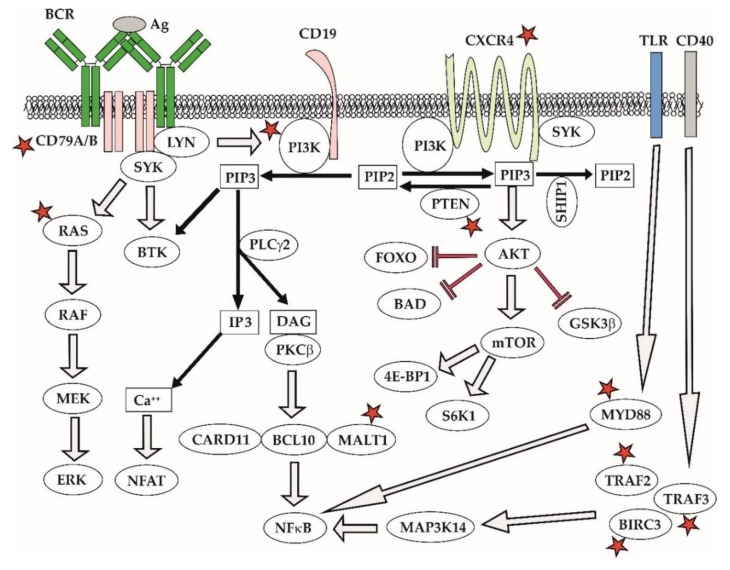
**B cell receptor and PI3K signaling. Abbreviations:** AKT—protein kinase B/AKT, BCL10—B cell lymphoma 10, BCR—B-cell receptor signaling, BIRC3—Baculoviral IAP (inhibitor of apoptosis) Repeat Containing 3, BTK—Bruton tyrosine-kinase, CARD11—Caspase Recruitment Domain Family Member 11, CXCR4—C-X-C Motif Chemokine Receptor 4, DAG—diacylglycerol, ERK—extracellular signal-regulated kinase, FOXO—Forkhead Box O, GSK3β—glycogene synthase kinase 3 beta, LYN—Lck/Yes-Related Novel Protein Tyrosine Kinase, MALT1—Mucosa-Associated Lymphoid Tissue Lymphoma Translocation Protein 1, MAP3K14—Mitogen-Activated Protein Kinase Kinase Kinase 14, MEK—mitogen-activated protein kinase kinase, MYD88—Myeloid Differentiation Primary Response Gene 88, mTOR—mammalian target of rapamycine, NFAT—nuclear factor of activated T-cells, NFκB—nuclear factor kappa B, PI3K—phosphatidylinositol 3-kinase, TLR—Toll-like receptor, PIP3—phosphoinositide 3,4,5-triphosphates, PIP2—phosphoinositide 4,5-diphosphosphate, PLCγ2—phospholipase C gamma 2, PTEN—phosphatase and tensin homolog, IP3—inositol 1,4,5-trisphosphate, RAF—RAF kinase, RAS—Raus sarcome oncogene, SHIP1—Src homology 2 (SH2) domain containing inositol polyphosphate 5-phosphatase 1, SYK—spleen tyrosine-kinase, S6K1—S6 kinase 1, TRAF2/3—Tumor Necrosis Factor Type 2 Receptor Associated Protein 2/3, 4E-BP—4E-binding protein. Asterisks highlight genes recurrently found in patients with lymphoma relapse after failure of ibrutinib.

Ibrutinib blocks BCR signaling, thereby depriving leukemia and lymphoma cells from vital prosurvival triggers [121,122]. Ibrutinib and next-generation BTK inhibitors (acalabrutinib, zanubrutinib) can induce a so-called compartment shift of leukemia and lymphoma cells causing egress of malignant lymphocytes deprived of the critical prosurvival signaling from the infiltrated lymph nodes and extra-nodal sites to peripheral blood where the cells eventually die off [123]. Inherent resistance to ibrutinib was observed in approximately 1/3 of MCL patients. Several molecular mechanisms of resistance to BTK inhibitors have been reported in MCL [124,125]. In contrast to chronic myelogeneous leukemia on imatinib therapy, mutations of BTK, e.g., C481S, are surprisingly uncommon in MCL [126]. The data suggest that acquired resistance to BTK inhibitors is mediated by aberrant activation of alternative prosurvival signaling pathways [124,127]. Mutations of CARD11, MALT1, TRAF2/3, or BIRC3 lead to aberrant activation of classical or alternative NFκB pathway (Figure 7) [128]. In addition, acquired resistance to ibrutinib has been repeatedly associated with enhanced activity of the PI3K–AKT pathway, caused, e.g., by downregulation of PTEN phosphatase and FOXO3a [127]. Notably, the AKT inhibitor MK2206 and the exportin 1 inhibitor selinexor both synergized with ibrutinib and were able to overcome the acquired resistance to ibrutinib [129,130]. Ibrutinib-resistant MCL cells also appear to be more dependent on BCL2 antiapoptotic signaling and the combination of ibrutinib and venetoclax has emerged as a highly promising combination [131]. Mutations of TP53 and NSD2 have developed in 3 out of 4 patients with blastoid transformation of MCL on ibrutinib [132]. In addition to somatic mutations, complex adaptive changes in response to microenvironmental factors play an important role in contributing to acquired ibrutinib resistance [126]. These results underline the need for better understanding of molecular mechanisms of resistance that would reveal relevant druggable targets and guide the design of rational treatment combinations.

In WM, mutations of CXCR4 and MYD88 correlate with the resistance and sensitivity to ibrutinib, respectively. Activating somatic mutations of *MYD88^L265P^*, present in >95% of WM patients, trigger oncogenic NFkappaB signaling predominantly through BTK, the reason why ibrutinib is an effective approach for the elimination of WM cells with *MYD88^L265P^* mutation. In contrast, WM patients with wild-type MYD88^wt^ usually harbor activating mutations of the NFkappaB pathway downstream of BTK, which is associated with ibrutinib resistance. Approximately one third of WM patients with *MYD88^L265P^* develop subclonal mutations of the chemokine receptor *CXCR4*^S338X^ that promote PI3K–AKT and ERK cascades, which are responsible for the acquired ibrutinib resistance (Figure 7) [133,134]. 

## 7. Resistance to Idelalisib and Other PI3K Inhibitors

Disruption of class I PI3K pathway is a hallmark of many lymphoproliferative disorders, especially of indolent B-cell lymphomas, DLBCL, and CLL. Class I PI3K has four isoforms (alpha, beta, gamma, delta), which phosphorylate their downstream target, a phosphoinositide 4,5-diphosphate (PIP2), into phosphoinositide 3,4,5-triphosphates (PIP3). PIP3 in turn activates several key prosurvival cascades including phospholipase C gamma or BTK. PI3Kdelta and gamma isoforms are particularly important for B- and T-cell functions, respectively. Together with BCR signaling, PI3K mediates critical signals from the tumor microenvironment. PI3K–AKT–mTOR signaling belongs to master regulators of cell energy/metabolic pathways and autophagy. PI3K signaling can rescue BCR signaling both in BCR knock-out mice and in patients on BTK inhibitors. Aberrant overactivation of PI3K–AKT was associated with resistance to BTK inhibitors [130]. Key negative regulators, a phosphatase and tensin homolog (*PTEN*) or *SHIP1*, are often deleted or inhibited in malignant B-cells. 

Mechanisms of resistance to PI3K inhibitors remain poorly understood. Insulin-like growth factor 1 receptor (IGF1R) was associated with intrinsic resistance to idelalisib in tumor cells [135]. IGF1R, associated with trisomy 12 in CLL cells, represents a druggable target offering potential combinatorial and salvage treatment [136]. IL6-triggered Janus kinase(JAK)- signal transducer and activator of transcription (STAT) cascade was reported as a critical molecular mechanism underlying resistance of lymphoma cells to copanlisib and duvelisib [137]. One of the strategies to avoid acquired resistance and to increase the clearance of malignant lymphocytes is the combinations of PI3K inhibitors with other anti-lymphoma agents, including anti-CD20 antibodies, BTK inhibitors, or BCL2 inhibitors [138,139].

## 8. Resistance to Venetoclax

**Figure 8:**
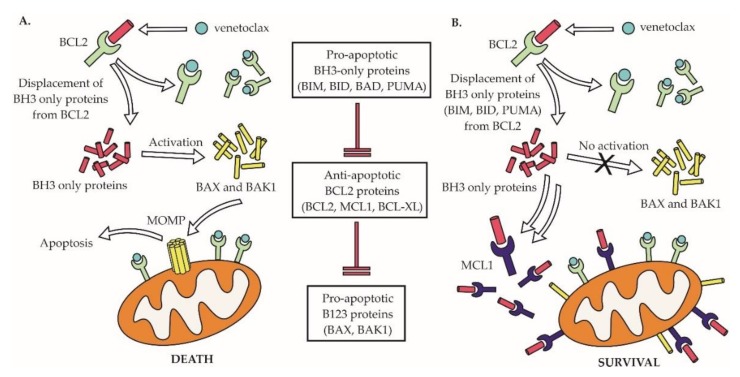
Overexpression of MCL1 is associated with acquired resistance of MCL cells to venetoclax**. A**. Mode of action of venetoclax in venetoclax-sensitive lymphoma cells. **B**. Overexpression of MCL1 leads to resistance to venetoclax. BH3—BCL2 homology 3.

As discussed above, the disruption of the anti-apoptotic B-cell lymphoma 2 (BCL2) molecule is a hallmark of the majority of non-Hodgkin lymphomas. Venetoclax (ABT-199) was the first-in-class BCL2 inhibitor approved for the treatment of cancer, for patients with R/R CLL [140,141]. In NHLs, venetoclax appears to be particularly effective in MCL and is in advanced clinical testing (phase 3 trials) in patients with DLBCL [142]. Venetoclax belongs to BCL2 homology 3 (BH3) mimetics that trigger cancer cell apoptosis by displacing proapoptotic BCL2 proteins, including BIM from the anti-apoptotic BCL2 protein (Figure 8). We and others have demonstrated that the MCL1/NOXA complex plays a pivotal role in mediating acquired venetoclax resistance in MCL, and that such a resistance can be overcome by concurrent therapy with the MCL1 inhibitor S63845 [106,143,144]. The disruption of other BCL2 family members, including the upregulation of BCL-XL, loss of BIM, or mutation of BAX were all associated with acquired venetoclax resistance [145,146,147]. Mutations of the BH3 domain of *BCL2* gene responsible for venetoclax binding (e.g., G101V and D103Y) were found in CLL patients with acquired resistance to venetoclax therapy [148]. Dynamic changes of lymphoma cells in response to tumor microenvironment were repeatedly associated with venetoclax resistance [145]. Recently, complex transcriptional reprogramming with alteration of cell energy/metabolic pathways was identified as a key contributor to venetoclax resistance in lymphoma cells [149].

## 9. Conclusions

In the last two decades, our knowledge of the biology of lymphomas and landscape of therapeutic options for patients with these lymphoid malignancies significantly increased. As a consequence, the percentage of patients that can be cured with currently available treatments is permanently growing. Importantly, for patients with relapsed lymphomas, several novel targeted agents and new treatment modalities have been introduced into clinical practice, while many more are currently tested in numerous clinical trials. In some lymphoma subtypes, we have already entered the era of risk-stratified, patient-tailored therapy based on molecular/cytogenetic prognostic markers. A better understanding of mechanisms responsible for disease recurrence, including the clonal evolution or lymphoma cell plasticity, will, in the near future, lead to the discovery and clinical testing of other effective targeted drugs, which, together with synthetic immunotherapy and CAR T-cell technology, might already, by the end of the upcoming decade, lead to the eradication of diseases that would even today be considered treatment-refractory.

## Abbreviations

ADC	Antibody-drug conjugate
ADCC	Antibody-dependent cell-mediated cytotoxicity
ALCL	Anaplastic large cell lymphoma
AlloSCT	Allogeneic stem cell transplantation
AMPK	AMP-activated protein kinase
Ara-C	Cytarabine
ASCT	Autologous stem cell transplantation
ATM	Ataxia teleangiectasia mutated
BCL2	B cell lymphoma 2
BCR	B-cell receptor
BH3	BCL2 homology 3
BL	Burkitt lymphoma
BMI-I	B-cell specific Moloney murine leukemia virus integration site 1
BTK	Bruton tyrosine-kinase
CAR	Chimeric antigen receptor
CDC	Complement-dependent cytotoxicity
CDK	Cyclin-dependent kinase
CLL	Chronic lymphocytic leukemia
CR	Complete remission
DCL	Deoxycytidine kinase
DDR	DNA damage response
DLBCL	Diffuse large B-cell lymphoma
HDT	High-dose therapy
EMEA	European Medicines Agency
FDA	Federal Drug Administration
FL	Follicular Lymphoma
MALT	Mucosa-associated lymphoid tissue
MCL	Mantle cell lymphoma
MMAE	Mono-methyl-auristatin E
MZL	Marginal zone lymphoma
MRD	Minimal residual disease
MTOR	Mammalian target of rapamycine
NFkappaB	Nuclear factor kappa B
NHL	Non-Hodgkin lymphoma
NK	Natural killer (cells)
ORR	Overall response rate (=complete and partial remissions)
OS	Overall survival
PARP1	poly(ADP)ribose-polymerase 1
PD(L)	Programmed cell death (ligand)
PFS	Progression-free survival
PI3K	Phosphoinositide-3 kinase
PIP2	phosphoinositide 4,5-diphosphate
PIP3	phosphoinositide 3,4,5-triphosphates
PLC	Phospholipase C
PPP	Pentose phosphate pathway
PR	Partial remission
PTEN	Phosphatase and tensin homolog
R-CHOP	Rituximab + cyclophosphamide + doxorubicin + vincristine + prednisone
R-DHAP	Rituximab + dexamethasone + high-dose cytarabine + cisplatin
RM	Rituximab maintenance
SHIP1	Src homology 2 containing inositol 5′ polyphosphatase 1
